# Autoimmune Diseases in Patients With Myotonic Dystrophy Type 2

**DOI:** 10.3389/fneur.2022.932883

**Published:** 2022-07-18

**Authors:** Stojan Peric, Jelena Zlatar, Luka Nikolic, Vukan Ivanovic, Jovan Pesovic, Ivana Petrovic Djordjevic, Svetlana Sreckovic, Dusanka Savic-Pavicevic, Giovanni Meola, Vidosava Rakocevic-Stojanovic

**Affiliations:** ^1^Neurology Clinic, University Clinical Center of Serbia, Belgrade, Serbia; ^2^Faculty of Medicine, University of Belgrade, Belgrade, Serbia; ^3^Center for Human Molecular Genetics, Faculty of Biology, University of Belgrade, Belgrade, Serbia; ^4^Cardiology Clinic, University Clinical Center of Serbia, Belgrade, Serbia; ^5^Anaesthesiology Clinic, University Clinical Center of Serbia, Belgrade, Serbia; ^6^Department of Neurorehabilitation Sciences, Casa Di Cura del Policlinico, Department of Biomedical Sciences for Health, University of Milan, Milan, Italy

**Keywords:** myotonic dystrophy type 2 (DM2), autoimmune diseases, antinuclear antibodies, antineutrophil cytoplasmic antibodies, Hashimoto autoimmune thyroiditis

## Abstract

**Introduction:**

Myotonic dystrophy type 2 (DM2) is a rare autosomal dominant multisystemic disease with highly variable clinical presentation. Several case reports and one cohort study suggested a significant association between DM2 and autoimmune diseases (AIDs).

**Aim:**

The aim of this study is to analyze the frequency and type of AIDs in patients with DM2 from the Serbian DM registry.

**Patients and Methods:**

A total of 131 patients with DM2 from 108 families were included, [62.6% women, mean age at DM2 onset 40.4 (with standard deviation 13) years, age at entering the registry 52 (12.8) years, and age at analysis 58.4 (12.8) years]. Data were obtained from Akhenaten, the Serbian registry for DM, and through the hospital electronic data system.

**Results:**

Upon entering the registry, 35 (26.7%) of the 131 patients with DM2 had AIDs including Hashimoto thyroiditis (18.1%), rheumatoid arthritis, diabetes mellitus type 1, systemic lupus, Sjogren's disease, localized scleroderma, psoriasis, celiac disease, Graves's disease, neuromyelitis optica, myasthenia gravis, and Guillain-Barre syndrome. At the time of data analysis, one additional patient developed new AIDs, so eventually, 36 (28.8%) of 125 DM2 survivors had AIDs. Antinuclear antibodies (ANAs) were found in 14 (10.7%) of 63 tested patients, including 12 without defined corresponding AID (all in low titers, 1:40 to 1:160). Antineutrophil cytoplasmic antibodies (ANCAs) were negative in all 50 tested cases. The percentage of women was significantly higher among patients with AIDs (82.9% vs. 55.2%, *p* <0.01).

**Conclusion:**

AIDs were present in as high as 30% of the patients with DM2. Thus, screening for AIDs in DM2 seems reasonable. Presence of AIDs and/or ANAs may lead to under-diagnosis of DM2.

## Introduction

Myotonic dystrophy type 2 (DM2) is a multisystem disorder, inherited in an autosomal-dominant pattern, and presents with a steadily progressive mild proximal muscle weakness mainly in the lower limbs, fluctuating myotonia, muscle pain, cataracts, and cardiac, endocrine, and metabolic abnormalities, most notably glucose and lipid metabolism impairments (1). There have also been reports of brain involvement in DM2 (2). The overwhelming number of issues patients with DM2 face makes a tremendous impact on their everyday life and poses diagnostic and treatment challenges for practitioners. Average diagnostic delay for DM2 is 14 years, and DM2 still remains underdiagnosed (3).

DM2 is caused by tetranucleotide (CCTG) repeat expansion in intron 1 of the *CNBP* gene on chromosome 3 (4). Mutation is in the region that is transcribed into mRNA but not translated into protein. Thus, CCUG repeat sequences accumulate in nuclei and exert a toxic effect mostly by dysregulation of RNA-binding proteins including muscleblind-like proteins (MBNL) (5). This results in increased expression of less functional, embryonic isoforms of different proteins in adult tissues (1, 4). Therefore, the pathophysiology of DM2 is truly multifaceted and complex.

Around 75% of patients with DM2 have hypogammaglobulinemia of IgG and IgM subtypes, with normal levels of IgA on serum electrophoresis (6). However, these findings have not been associated with any clinical abnormalities so far. Tieleman et al. conducted a study on 28 patients with DM2 and concluded that autoimmune diseases (AIDs), antinuclear antibodies (ANAs), and rheumatoid factor (RF) were more frequent in patients with DM2 (21%) than in patients with DM1 and the general population (2%) (7). The AIDs diagnosed in patients with DM2 included rheumatoid arthritis, Sjogren's syndrome, Churg-Strauss syndrome, anterior uveitis, autoimmune hepatitis, and aplastic anemia.

The aim of our research was to establish the frequency and type of AIDs in a large cohort of Serbian patients with DM2, as well as to analyze potential associations between AIDs and sociodemographic/clinical features of patients with DM2.

## Materials and Methods

Data were collected from *Akhenaten*, the Serbian registry for myotonic dystrophies, founded in 2008 (8). The formation of the registry was approved by the Ethics Committee of the Faculty of Medicine, University of Belgrade. All the patients signed the informed consent to share their anonymous data in the registry, as well as to use the data for secondary academic purposes. Patients' data entered into the registry were based on a clinical questionnaire performed by a neuromuscular specialist. In all the patients, the clinical diagnosis of DM2 was confirmed by real-time polymerase chain reaction (RP-PCR) showing an increased number of tetranucleotide repeats in the *CNBP* gene (9). The registry also included results from hormonal, biochemical, and immune-serological testing at the time of entering the registry. Additionally, we supplemented our database with data from the hospital electronic medical records of patients with DM2, with the aim of uncovering AIDs diagnosed after their entry into the registry. The institution review board approved the design of the study.

Sociodemographic parameters of interest for our study were gender, age at onset of DM2 symptoms, age at entering the registry, and current age. Analyzed clinical parameters included presence of hand grip myotonia, thenar percussion myotonia, and muscle strength. Limb muscle strength was estimated using the Medical Research Council (MRC) 0- to 5-point scale. The following muscles were tested: shoulder abductors and adductors, elbow flexors and extensors, wrist and finger flexors and extensors, hip flexors, extensors, abductors, and adductors, knee flexors and extensors, and plantar and dorsal ankle and toe flexors. The strength of the weakest muscle of the proximal and distal upper and lower limbs was added together to form the MRC sum score representing overall muscle strength (10). Needle electromyography (EMG) was performed to detect myopathic motor units and myotonia in multiple muscles (at least in deltoid, biceps brachii, flexor digitorum profundus, vastus, and tibialis anterior), while nerve conduction studies (NCSs) were conducted to detect the presence of polyneuropathy. Cardiac function was evaluated by clinical examination, blood pressure measurement, electrocardiography (ECG), with particular attention to the presence of conduction defects and arrhythmias, and echocardiography. Positive medical history for an implanted pacemaker was also noticed. The patients underwent detailed laboratory investigations, including serum glucose and insulin levels, HOMA-IR (Homeostatic Model Assessment of Insulin Resistance), oral glucose tolerance tests, levels of free T4 and TSH, and presence of thyroid-specific autoantibodies (anti-thyroid peroxidase, TPO, and anti-thyroglobulin, Tg). The patients underwent an ophthalmological examination for the presence of cataracts. They were also asked about infertility and spontaneous abortions. Data on use of different medications were also added at the time of entering the registry.

Upon entering the registry, all the patients with DM2 were examined in detail about their previous diseases, including AIDs. For the purpose of our investigation, AIDs were defined as conditions brought about by failure of the immune system in distinguishing self from non-self (11). These included but were not limited to connective tissue diseases, systemic vasculitis, and dermatological, endocrine, neurological, gastrointestinal, hematological, and ophthalmological disorders. Unfortunately, data on age at onset of AIDs were not collected. It is important to note that immune serology test was not a routine part of clinical investigation of patients with DM2, and it was available only for those that were investigated through the inpatient unit or the Day Hospital of the Neurology Clinic. The reason for performing immune serology was not suspicion of any immune-mediated disease, but this screening is regularly performed on all patients with myopathies. History and clinical and laboratory data on AIDs were critically supported by investigation of the patients' electronic medical records that comprised pieces of information after entering the registry.

Means, standard deviations, and proportions were used as methods of descriptive statistics. Chi square test, Mann-Whitney U test and Student's *t*-test were conducted to examine differences between two groups. The level of statistical significance was set at *p* <0.05.

## Results

**Figure 1:**
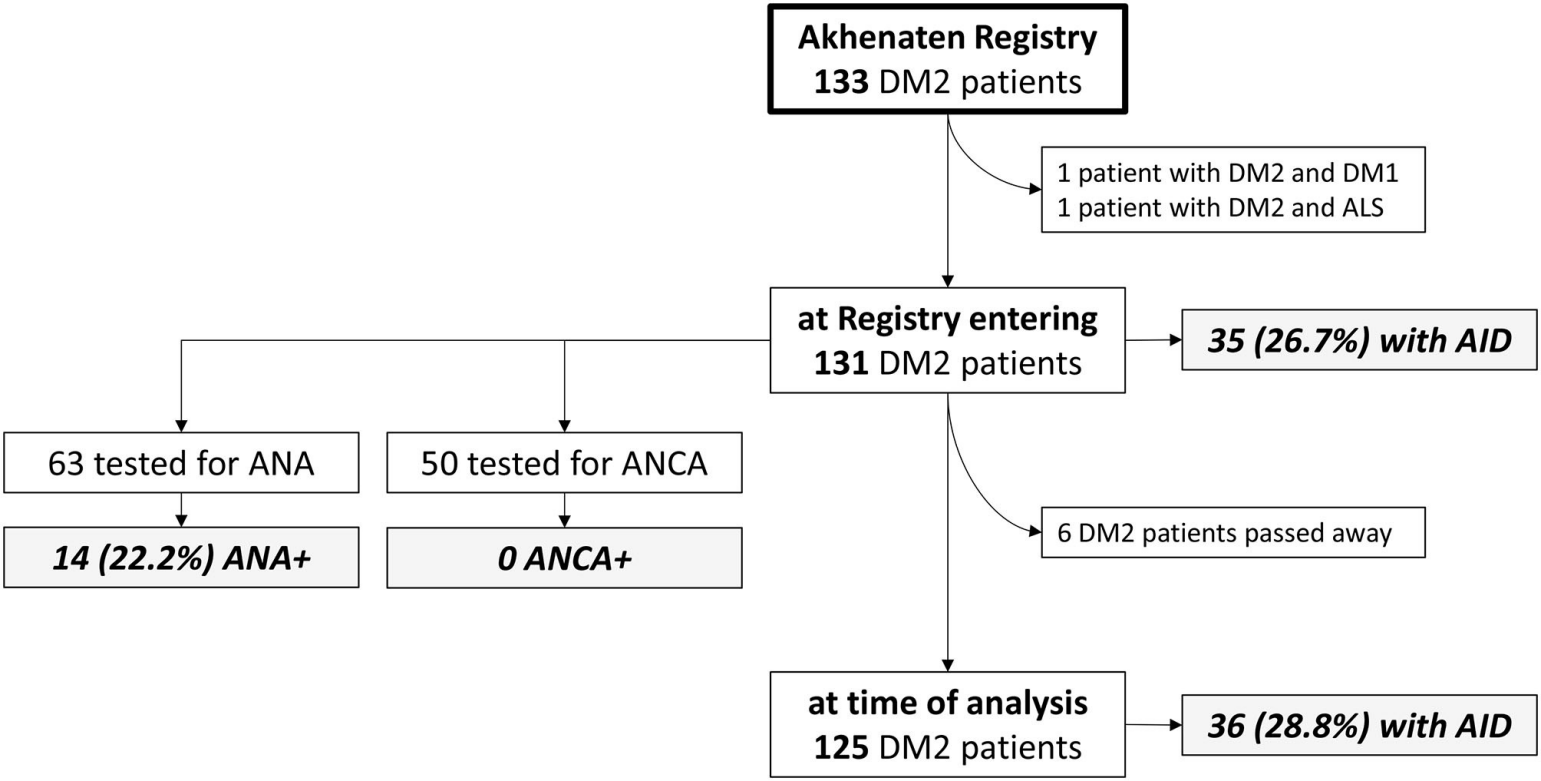
Flowchart representing the analyzed DM2 cohort and frequency of autoimmune diseases. AID, autoimmune disease; ALS, amyotrophic lateral sclerosis; ANA, antinuclear antibody; ANCA, antineutrophilic antibody; DM1, myotonic dystrophy type 1; DM2, myotonic dystrophy type 2.

After we excluded double entries for same patients and patients with other significant diseases (one patient had both DM2 and DM1, and one patient had DM2 and amyotrophic lateral sclerosis), the *Akhenaten* registry yielded 131 patients from 108 families for our cohort (Figure 1). In this cohort, 82 (62%) of the patients were women (n = 82), mean age at disease onset was 40.4 ± 13 years, age at registry entry was 52 ± 12.8 years, and age at the time of the study was 58.4 ± 12.8 years (Table 1). Muscle weakness was most pronounced in the proximal lower limbs. Cataracts were present in 71.6% of the patients with DM2, hypertension in 52.7%, ECG abnormalities in 12.9% (rhythm other than sinus, PR interval of 240 ms or more, QRS duration of 120 ms or more, second-degree or third-degree AV block), and echocardiographic abnormalities in 29.6% (most common was prolonged relaxation of the left ventricle). Long-term cardiac monitoring such as Holter/event monitors would be a better way to probe conduction defects and dysrhythmias in DM2, but these data were available only for the minority of our patients with DM2. Spontaneous abortions were reported by 32.5% of the female patients. With regard to laboratory investigations, insulin resistance was present in 24.6% of the patients, and diabetes in 22.8%. Hypothyroidism was noticed in 16.2% of the patients and hyperthyroidism in 3.8% (Table 1).

**Table 1:** Sociodemographic, clinical, and laboratory features of patients with DM2.

**Feature**	**Number (%) of patients with available data**	**Mean ± SD or number (%) of patients**
Female gender	131 (100%)	82 (62%)
Age at entry	131 (100%)	52.0 ± 12.8
Age at onset	131 (100%)	40.4 ± 13.0
Current age	131 (100%)	58.4 ± 12.8
Limb muscle strength	131 (100%)	
Proximal upper limb	131 (100%)	4.6 ± 0.5
Distal upper limb	131 (100%)	4.5 ± 0.7
Proximal lower limb	131 (100%)	4.0 ± 0.7
Distal lower limb	131 (100%)	4.5 ± 0.7
MCR	131 (100%)	17.1 ± 3.4
Actionhand grip myotonia	101 (77.1%)	46 (45.5%)
Percussion hand myotonia	100 (76.3%)	60 (60%)
EMG/NCS	109 (83.2%)	
Myopathic MG changes	109 (83.2%)	87 (79.8%)
Myotonic discharges	109 (83.2%)	83 (76.1%)
Polyneuropathy	105 (80.2%)	26 (24.8%)
Cataracts	116 (88.5%)	83 (71.6%)
Hypertension	131 (100%)	69 (52.7%)
ECG abnormality	93 (71.0%)	12 (12.9%)
Implanted pacemaker	126 (96.2%)	4 (3.1%)
Echocardiographic abnormality	81 (61.8%)	24 (29.6%)
Infertility	93 (71.0%)	9 (9.7%)
Spontaneous abortion	40 (30.5%)	13 (32.5%)
Glucose metabolism	114 (87.0%)	
Normal	114 (87.0%)	60 (52.6%)
Insulin resistance/glucose intolerance	114 (87.0%)	28 (24.6%)
Diabetes	114 (87.0%)	26 (22.8%)
Thyroid status	105 (80.2%)	
Hypothyroid	105 (80.2%)	17 (16.2%)
Normal	105 (80.2%)	86 (81.9%)
Hyperthyroid	105 (80.2%)	4 (3.8%)

In our DM2 cohort, 35 patients (26.7%) reported that they had been diagnosed with an AID before entering the registry (Table 2). The most diagnosed AID in our cohort was Hashimoto thyroiditis with positive anti-TPO and/or anti-Tg antibodies diagnosed by an endocrinologist, which was found in 23 (17.6%) patients. Excluding Hashimoto thyroiditis, we were left with 15 (11.5%) patients with other AIDs. Type 1 diabetes mellitus occurred in 2 (1.5%) patients, and all of the other AIDs were found in single patients. The diseases included rheumatoid arthritis, systemic lupus, Sjogren's syndrome, localized scleroderma, psoriasis, celiac disease, Graves's disease, neuromyelitis optica with positive antibodies against aquaporin 4, and myasthenia gravis. Of note, four patients had been diagnosed with two AIDs: Hashimoto's disease and lupus, or psoriasis, or rheumatoid arthritis, or type 1 diabetes. One additional patient had Graves' disease and Raynaud phenomenon.

**Table 2:** Autoimmune diseases and auto-antibodies in patients with DM2.

**Feature**	**Number (%) of patients**
Presence of AID at entering Registry	35 (26.7%) of 131 patients
Presence of AID at entering Registry	15 (11.5%) of 131 patients
when Hashimoto thyroiditis is excluded	
Presence of AID at time of analysis	36 (28.8%) of 125 patients
Presence of ANA at entering Registry	14 (22.2%) of 63 tested patients
Presence of ANCA at entering Registry	0 (0%) of 50 tested patients

Between entry into the registry and the time of analysis (median of 6.5 years), six of the patients with DM2 have passed away: three due to malignancy (colon carcinoma, leukemia, and brain tumor), the others due to influenza, ileus, and complications of neuromyelitis optica. Of the remaining 125 patients, one was additionally diagnosed with AID so eventually 36 (28.8%) of 125 DM2 survivors had AIDs. The new AID diagnosis was Guillain-Barre syndrome (Table 2).

The most assayed autoantibodies in our cohort were antinuclear antibodies (ANAs) tested in 63 (48.1%) of the patients (71.4% women, age 52.4 ± 11.1 years, age at onset 39.4 ± 12.5 years, MRC 17.5 ± 1.8) and antineutrophil cytoplasmic antibodies (ANCAs) tested in 50 (38.2%) of the patients(74% women, age 51.5 ± 11.3 years, age at onset 39.4 ± 12.1 years, MRC 17.6 ± 1.8). Cohorts tested for ANAs or ANCAs did not differ in their sociodemographic features and muscle strength compared to the whole DM2 group. The results of immune serology test are presented in Figure 1 and Table 2. We found that 14 (22.2%) of the tested patients were positive for ANAs, including 12 patients without defined corresponding AID (all in low titers, 1:40 to 1:160). No patients were found to have ANCAs. The other assayed autoantibodies were not statistically analyzed because of insufficient number of patients.

Finally, we tested for associations between patient demographics and clinical parameters and their autoimmune status. The percentage of women was significantly higher among patients with AIDs (82.9 vs. 55.2%, *p* < 0.01). The patients with AIDs were less likely to have myopathic changes on needle EMG (66.7 vs. 84.8%, *p* < 0.05). Also, 50% of the patients with AIDs had some degree of thyroid dysfunction compared to only 6.7% without it (*p* < 0.01). We also found that patients with positive ANA had myopathic changes on EMG in only 61% of patients compared to 88.9% of those without ANA (*p* < 0.05). We found no other significant correlations.

## Discussion

Our analysis suggests that AIDs may be more common in patients with DM2; 27% of the patients already had an AID before entering the DM2 registry. A high percentage of AIDs in DM2 (43%) was also found in a Dutch cohort reported by Tieleman et al. (7). However, the design of the two studies is different. Our data were extrapolated from the nationwide database, while the Dutch cohort consisted of patients with DM2 age- and sex-matched with DM1 controls, and all were interviewed with a standardized clinical questionnaire. An additional difference is that the Dutch study included B12 deficiency as a potential AID even without measurement of specific autoantibodies. When B12 deficiency is excluded, the percentage of patients with AID in the Dutch study fell from 43 to 21%, which is closer to our findings. It is important to note that upon exclusion of B12 deficiency, the most common AIDs in the Dutch patients with DM2 were hypothyroidism and hyperthyroidism like in our cohort where Hashimoto's thyroiditis was the most common AID. Furthermore, when comparing the Serbian and Dutch cohorts, Graves' disease, Sjogren's syndrome, and rheumatoid arthritis appeared in both. The AIDs seen exclusively in the Dutch patient sample included aplastic anemia, uveitis anterior, Churg-Strauss syndrome, and autoimmune hepatitis, while the Serbian database included myasthenia gravis, neuromyelitis optica, celiac disease, and psoriasis.

These findings have profound diagnostic implications, since symptoms of DM2 and a variety of AIDs often overlap, like in the case of Hashimoto or Graves that can give rise to myopathy and myotonia (12, 13). This can result in earlier diagnosis of DM2 on the one hand or lead to DM2 being completely missed, since myotonia and myopathy might be interpreted as part of a thyroid disease. A similar error may occur when practitioners arrive at a diagnosis of connective tissue disorders, with weakness, myalgia, fatigue, gastrointestinal issues collectively being attributed to AIDs instead of DM2 (14). Patients that suffer from a neurological AID in the setting of DM2 are of particular interest. Two case reports involving a patient with DM2 and myasthenia gravis showed how, in this rare scenario, AID diagnosis opens a path to more successful treatment (15, 16). Several other case reports of DM2 with an autoimmune disease have been published so far (17–24, Table 3). Precise diagnosis helps to ensure that detrimental immuno-suppressive drugs are not unnecessarily utilized in patients with DM2, since they can accelerate the progression of cataracts and worsen insulin resistance (25, 26).

**Table 3:** Previous literature data on AIDs in DM2.

**Author (year)**	**Article title**	**Number of patients with concomitant AIDs and AIDs type**
Bamberg et al. (16)	Coincidence of myasthenia gravis and myotonic dystrophy type 2	1 myasthenia gravis
Tieleman et al. (7)	Strong association between myotonic dystrophy type 2 and autoimmune diseases	2 hypothyroidism; 2 hyperthyroidism; 2 rheumatoid arthritis; 1 uveitis anterior; 1 autoimmune hepatitis; 1 Sjogren disease; 1 aplastic anemia; 1 Churg-Strauss syndrome
Sicureli et al. (17)	Myotonic dystrophy type 2 and autoimmune chronic gastritis: an incidental association	1 Hashimoto thyroiditis + idiopathic thrombocytopenic purpura + autoimmune chronic gastritis
Ehler et al. (18)	Myotonic dystrophy type 2 and multiple sclerosis: case report	1 multiple sclerosis
Nikolic et al. (15)	The coexistence of myasthenia gravis and myotonic dystrophy type 2 in a single patient	1 myasthenia gravis
Meyer et al. (19)	Eosinophilic myositis as first manifestation in a patient with DM2 CCTG expansion mutation and rheumatoid arthritis	1 eosinophilic myositis + rheumatoid arthritis
Rakocevic-Stojanovic et al. (20)	Neuromyelitis Optica in a Patient from Family with both Myotonic Dystrophy Type 1 and 2	1 neuromyelitis optica
Damen et al. (21)	Graves' disease and celiac disease in a patient with myotonic dystrophy type 2	1 Graves' disease + celiac disease
Karatzikou et al. (22)	White matter hyperintensities in myotonic dystrophy type 2: not always another expression of the disease	1 multiple sclerosis
Schoser et al. (23)	Self-diagnosis of a Triple Trouble	1 myasthenia gravis + acquired rippling muscle disease
Gelibter et al. (24)	Neuromyelitis optica and myotonic dystrophy type 2: a rare association with diagnostic implications	1 neuromyelitis optica

*AIDs, autoimmune diseases*.

The process of establishing a correct diagnosis is complicated by the fact that a large percentage of patients with DM2 test positive for autoantibodies such as ANAs (roughly 61% in the Dutch study and 22% in our Serbian database) without accompanying symptoms of AIDs. This is not particularly surprising, as ANAs in low titers may appear in up to 40% of healthy people, while AIDs develop in only 5-7% of the general population, 80% of whom are women (27, 28). Similarly, we found female predominance in our patients with DM2 and AIDs. Reasons behind the difference in AIDs between sexes are not yet completely understood, either in the general population or in DM2.

Interestingly, we have observed that the patients with DM2 and AIDs with or without ANAs often exhibit fewer signs of myopathic changes on needle EMG. Because of AID symptoms, patients may be caught and diagnosed with DM2 earlier in the disease course, i.e., before a myopathic pattern on EMG has a chance to develop.

The pathogenesis of AIDs in DM2 remains an intriguing question. As mentioned above, DM2 is caused by a repeat expansion sequence that is transcribed into RNA but not translated into protein, which dysregulates the homeostasis of RNA-binding proteins (especially the MBNL protein group) and leads to alternative proteins synthesis. These abnormalities in pre-mRNA processing support the theory of spliceopathy: an aberrant nuclear environment is giving rise to mis-spliced proteins (29). This would explain why the immune system, whose mechanisms of both central and peripheral tolerance rely heavily on specific molecular signaling pathways, may become dysregulated in such a cellular environment. On the other hand, Tieleman et al. (7) proposed another theory of origin: a separate gene flanking *CNBP* might be mutated and inherited more often together with mutated *CNBP* because of linkage disequilibrium. For example, the 3q13 chromosomal region is adjacent to the *CNBP* gene locus. It has been shown as a predisposing locus for several immune-mediated diseases including celiac disease and rheumatoid arthritis, which are both described in patients with DM2 (30, 31). This would explain why the association with AIDs is seen only with DM2 and not with DM1, which has an otherwise similar pathology.

The presence of AIDs in DM2 might be at least partially a product of epistasis. Epistasis is a phenomenon in which the effect of a gene mutation is dependent on the presence or absence of mutations in other (modifier) genes (32). Epistasis has been described in immune-mediated diseases in the context of interactions between different alleles of human-leukocyte antigen genes. A well-known example is that of the genetic predisposition for celiac disease: while HLA DQ-6.2, HLA 7.3 and HLA-DQ 2.5 seem to create a predisposition on their own, when inherited together, in the event of long-term high-dose gluten exposure, celiac disease may develop (33). Future sequencing of whole genomes of patients with DM2 might shed more light on this possibility. To understand better the autoimmunity in DM2, future studies should also focus on assays to measure B cell and T cell activity and interleukin pathways. Our study is lacking those measures and is only an introduction to understanding this issue in patients with DM2. The main limitations of our study are the retrospective nature of the analysis and lack of a control group.

## Conclusion

AIDs were present in as high as 30% of patients with DM2. Thus, screening for AIDs in DM2 seems reasonable. Presence of AIDs and/or ANAs may lead to underdiagnosis of DM2 in the general population.

## Data Availability Statement

The raw data supporting the conclusions of this article will be made available by the authors, without undue reservation.

## Ethics Statement

The studies involving human participants were reviewed and approved by University of Belgrade–Faculty of Medicine. The patients/participants provided their written informed consent to participate in this study.

## Author Contributions

SP, GM, and VR-S contributed to the concept or design of the study. JZ, LN, VI, SS, IP, and SP contributed to the collection of data. JZ, LN, VI, SP, JP, DS-P, GM, and VR-S contributed to the analysis and interpretation of data. JZ, LN, and VI produced the first draft of the paper based on input and direction from all authors. All authors provided input into subsequent drafts, reviewed and approved the final version for submission, had full access to the study design information, and had final responsibility for the decision to submit for publication.

## Funding

GM was supported by Fondazione Malattie Miotoniche (FMM), Milan, Italy.

## Conflict of Interest

The authors declare that the research was conducted in the absence of any commercial or financial relationships that could be construed as a potential conflict of interest.

## Publisher's Note

All claims expressed in this article are solely those of the authors and do not necessarily represent those of their affiliated organizations, or those of the publisher, the editors and the reviewers. Any product that may be evaluated in this article, or claim that may be made by its manufacturer, is not guaranteed or endorsed by the publisher.
